# DDX3X Biomarker Correlates with Poor Survival in Human Gliomas

**DOI:** 10.3390/ijms160715578

**Published:** 2015-07-09

**Authors:** Dueng-Yuan Hueng, Wen-Chiuan Tsai, Hsin-Ying Clair Chiou, Shao-Wei Feng, Chin Lin, Yao-Feng Li, Li-Chun Huang, Ming-Hong Lin

**Affiliations:** 1 Department of Neurological Surgery, Tri-Service General Hospital, National Defense Medical Center, No. 325, Section 2, Taipei 11490, Taiwan; E-Mails: phoenixchiou@gmail.com (H.-Y.C.C.); heisenber0930@gmail.com (S.-W.F.); 2 Department of Biochemistry, National Defense Medical Center, No. 325, Section 2, Taipei 11490, Taiwan; E-Mail: emily7781@hotmail.com; 3 Department of Pathology, Tri-Service General Hospital, National Defense Medical Center, Taipei 11490, Taiwan; E-Mails: drtsaiwenchuan@mail2000.com.tw (W.-C.T.); liyaofeng1109@gmail.com (Y.-F.L.); 4 Graduate Institute of Life Science, National Defense Medical Center, Taipei 11490, Taiwan; E-Mail: xup6fup@hotmail.com; 5 Department and Graduate Institute of Microbiology and Immunology, National Defense Medical Center, Taipei 11490, Taiwan; E-Mail: blimp1@hotmail.com.tw

**Keywords:** glioma, WHO grades, GEO profile, tissue microarray, DDX3X, RNA Helicase DDX3X

## Abstract

Primary high-grade gliomas possess invasive growth and lead to unfavorable survival outcome. The investigation of biomarkers for prediction of survival outcome in patients with gliomas is important for clinical assessment. The DEAD (Asp-Glu-Ala-Asp) box helicase 3, X-linked (DDX3X) controls tumor migration, proliferation, and progression. However, the role of DDX3X in defining the pathological grading and survival outcome in patients with human gliomas is not yet clarified. We analyzed the *DDX3X* gene expression, WHO pathological grading, and overall survival from de-linked data. Further validation was done using quantitative RT-PCR of cDNA from normal brain and glioma, and immunohistochemical (IHC) staining of tissue microarray. Statistical analysis of GEO datasets showed that DDX3X mRNA expression demonstrated statistically higher in WHO grade IV (*n* = 81) than in non-tumor controls (*n* = 23, *p* = 1.13 × 10^−1^^0^). Moreover, DDX3X level was also higher in WHO grade III (*n* = 19) than in non-tumor controls (*p* = 2.43 × 10^−5^). Kaplan–Meier survival analysis showed poor survival in patients with high DDX3X mRNA levels (*n* = 24) than in those with low DDX3X expression (*n* = 53) (median survival, 115 *vs.* 58 weeks, *p* = 0.0009, by log-rank test, hazard ratio: 0.3507, 95% CI: 0.1893–0.6496). Furthermore, DDX3X mRNA expression and protein production significantly increased in glioma cells compared with normal brain tissue examined by quantitative RT-PCR, and Western blot. IHC staining showed highly staining of high-grade glioma in comparison with normal brain tissue. Taken together, DDX3X expression level positively correlates with WHO pathologic grading and poor survival outcome, indicating that DDX3X is a valuable biomarker in human gliomas.

## 1. Introduction

Glioblastomas are the most frequent primary brain cancers with poor outcome due to their invasive characteristics despite maximal surgical resection and current chemo-radiotherapy [1,2]. Currently, the standard treatment includes maximal surgical resection and concurrent chemo-radiotherapy. However, the overall survival in patients with glioblastomas undergoing radiotherapy and temozolomide is 14.6 months on average [3]. The pathologic grading of gliomas classified by World Health Organization (WHO) grade I to IV [4]. Higher grade gliomas carry worse prognosis and increased fatality [5]. Therefore a genetic biomarker to differentiate the genetic expression in brain gliomas is crucial for prediction of clinical outcome [6].

The DEAD (Asp-Glu-Ala-Asp) box helicase 3, X-linked (DDX3X) or DEAD box polypeptide 3, X-linked, or DDX3X, belongs to ATP-dependent RNA helicase, which acts as a cofactor for exportin-1 (XPO1)-mediated nuclear export of partly spliced HIV-1 Rev RNAs [7]. DDX3X is known to participate in HIV-1 replication [8]. DDX3X related particularly with hepatitis C virus core protein lead to an alteration in intracellular location [9], and nucleic acid metabolism [10].

Activation of DDX3X enhances proliferation and malignancy [11]. DDX3X is expressed in many human malignancies, including cancers of the breast [12], lung [13], colon [14], liver [15], gallbladder [16], cervical cancer [17], and brain [17]. However, the role of DDX3X in determining the survival outcome and WHO pathologic grading of human gliomas is not yet clarified.

This study hypothesized that high-grade glioma overexpressed DDX3X. Based on this hypothesis, we intended to investigate if DDX3X expression is associated with the survival outcome and WHO pathologic grading in human gliomas. Analyses of the dataset from the Gene Expression Omnibus (GEO) profiles, a database that provides extensive genetic analyses of human gene expression and specific disease associations [5,18,19], show that DDX3X expression level closely correlates with WHO grading and poor survival outcome in patients with primary gliomas. The quantitative RT-PCR showed DDX3X mRNA expression is significantly higher in glioma cells compared to normal brain tissue. Western blot reveals DDX3X protein is highly produced in human glioma cell lines. Immunohistochemical (IHC) staining also demonstrates high affinity for high-grade glioma than normal brain tissue. Thus DDX3X can be a candidate biomarker in human gliomas.

## 2. Result

### 2.1. DDX3X Expression Positively Correlates with WHO Grading of Human Gliomas

**Figure 1:**
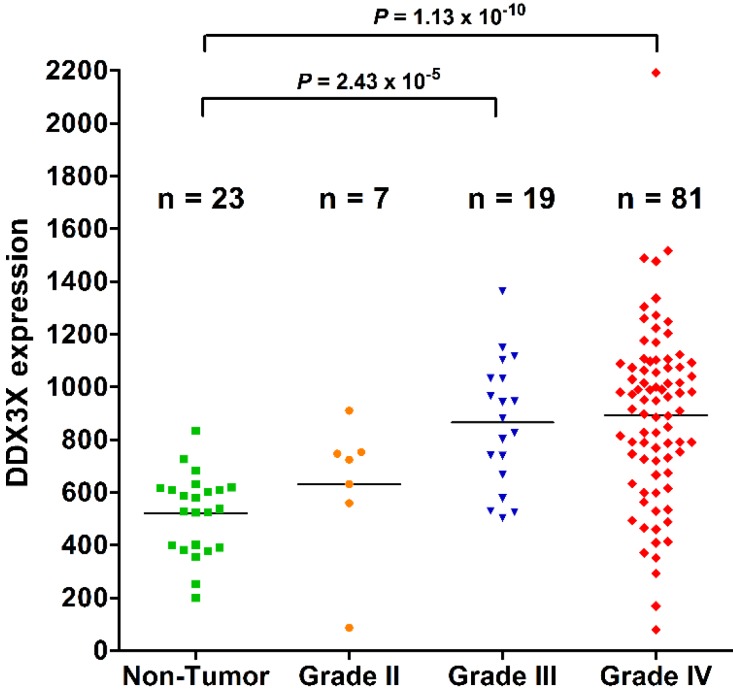
DDX3X expression positively correlates with WHO grading of human gliomas. The distributed plots show the *DDX3X* gene expression in low-grade glioma and non-tumor controls in comparison to those in high-grade gliomas. Increased DDX3X mRNA levels statistically correlates with WHO grades of human gliomas. The adjusted *p* value was calibrated between each group.

As shown in Figure 1, the DDX3X expression level was higher in WHO grade IV (*n* = 81) than in non-tumor controls (*n* = 23) (*p* = 1.13 × 10^−1^^0^). It is also higher in WHO grade III (*n* = 19) than in non-tumor controls (*p* = 2.43 × 10^−5^, *p* adjusted by Bonferroni method). These results show that high-grade gliomas overexpressed DDX3X.

### 2.2. DDX3X Expression Correlates with Poor Survival in High-Grade Gliomas

**Figure 2:**
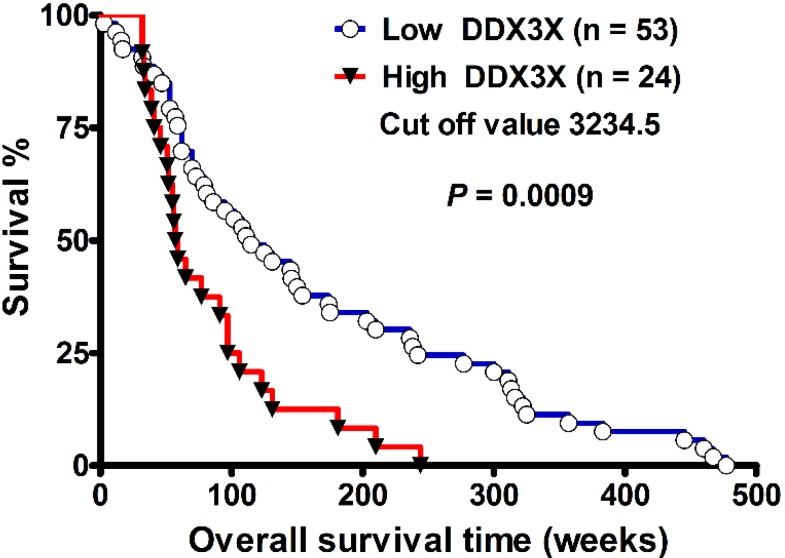
DDX3X expression correlates with poor survival in high-grade gliomas. The Kaplan–Meier survival curve demonstrated poor survival in those with high DDX3X (>3234.5) (*n* = 24) compared to those with low DDX3X (<3234.5) (*n* = 53) expression levels (median survival, 115 *vs.* 58 weeks, *p* = 0.0009, by log-rank test, hazard ratio: 0.3507, 95% CI: 0.1893–0.6496).

The demographic data including WHO pathologic grades, gender, age, and survival times were mentioned previously [18]. There were 21 patients (13 male, 8 female) in grade III population, and 56 patients (38 male, 18 female) in grade IV population. The age of grade III, and IV patients were 37.4 ± 9.9, and 48.5 ± 12.8 years old, respectively. The overall survival time in grade III, and IV was 227.0 ± 161.1, and 106.7 ± 82.0 weeks, respectively. As shown in Figure 2, the Kaplan–Meier survival curve of 77 patients with grade III and grade IV gliomas showed that patients with low DDX3X mRNA expression levels (*n* = 53) had better overall survival than those with high DDX3X mRNA expression levels (*n* = 24) (*p* = 0.0009, by log-rank test; 95% CI: 0.1893–0.6496, Ratio 0.3507). The cut-off value was set at 3234.5. The median survival interval in the high- and low-DDX3X expressions level was 58 and 115 weeks, respectively. In multivariate analysis, two variables showed significant correlation. These were DDX3X expression, and WHO pathologic grade. Survival rate was poor in the high DDX3X expression group (*p* = 0.02365, 1.879-fold hazard, 95% confidence interval: 1.088 to 3.246) than in the low DDX3X expression group after adjustments for WHO grade and age. Moreover, WHO grade IV showed a poorer survival rate than in the WHO grade III group (*p* = 0.00255, 2.767-fold hazard, 95% confidence interval: 1.429 to 5.361). In contrast, gender was excluded in multivariate analysis because it was not significant in univariate analysis (*p* = 0.269). The variable age did not reach statistical significance (*p* = 0.61747, 1.005-fold hazard, 95% confidence interval: 0.985 to 1.026).

### 2.3. DDX3X mRNA Expression Is Increased in Human Glioma Cells

**Figure 3:**
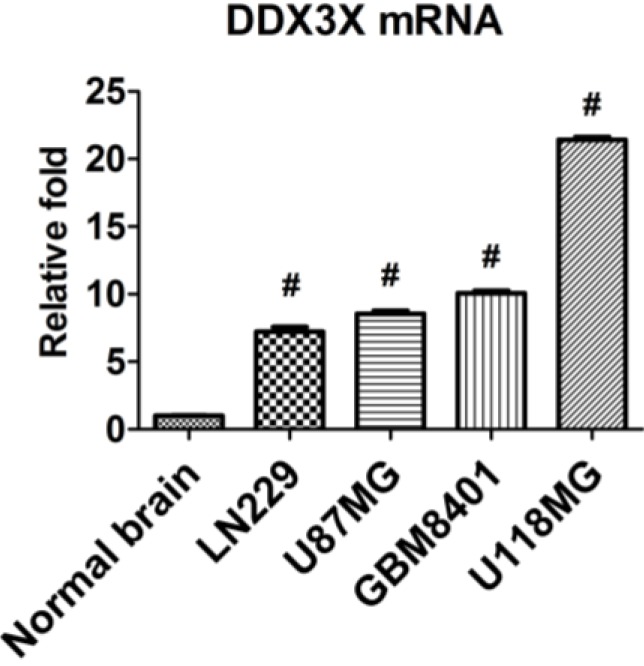
Expression of DDX3X mRNA in glioma cell lines and normal brain tissue. qRT-PCR was performed to examine DDX3X mRNA expression and the quantitative results are shown. The relative expressions were normalized with normal brain. Bars, mean ± SEM; # *p* < 0.001 showed significant differences. Data are representative of three independent experiments.

The DDX3X mRNA expressions were examined in glioma cells by q-RT PCR. The low-grade glioma cell line, LN229, and WHO grade IV glioma cell lines including U87MG, GBM8401, and U118MG were applied to compare DDX3X expression with normal brain. As shown in Figure 3, the expression of DDX3X was significantly increased in glioma cells in comparison with normal brain tissue.

### 2.4. DDX3X Protein Overexpresses in Human Glioma Cells

**Figure 4:**
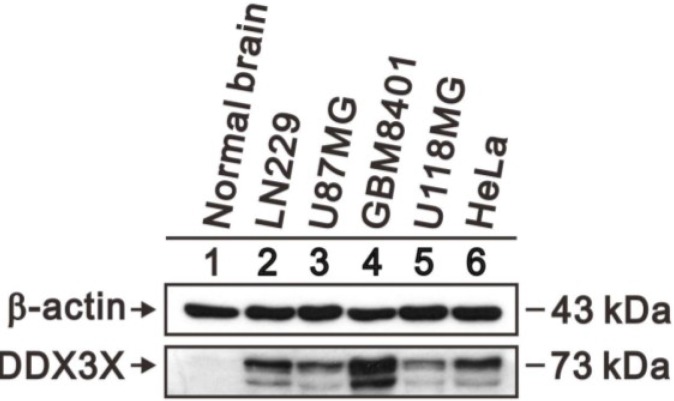
Expression of DDX3X protein in human glioma cell lines and normal brain tissue. Protein lysates of glioma cell lines, including LN229, U87MG, GBM8401 and U118MG, were applied to SDS-PAGE and Western blot analysis to quantitate DDX3X protein expression. HeLa cell was a positive control of DDX3X protein. β-actin served as a loading control.

DDX3X protein production in human glioma cell lines and normal brain tissue was quantitated by Western blot analysis. As shown in Figure 4, DDX3X protein significantly overexpressed in human glioma cell lines, including LN229, U87MG, GBM8401 and U118MG, compared to normal brain tissue. HeLa cell was a positive control for DDX3X protein production [17].

### 2.5. DDX3X Protein Production Overexpressed in Human High-Grade Gliomas

**Figure 5:**
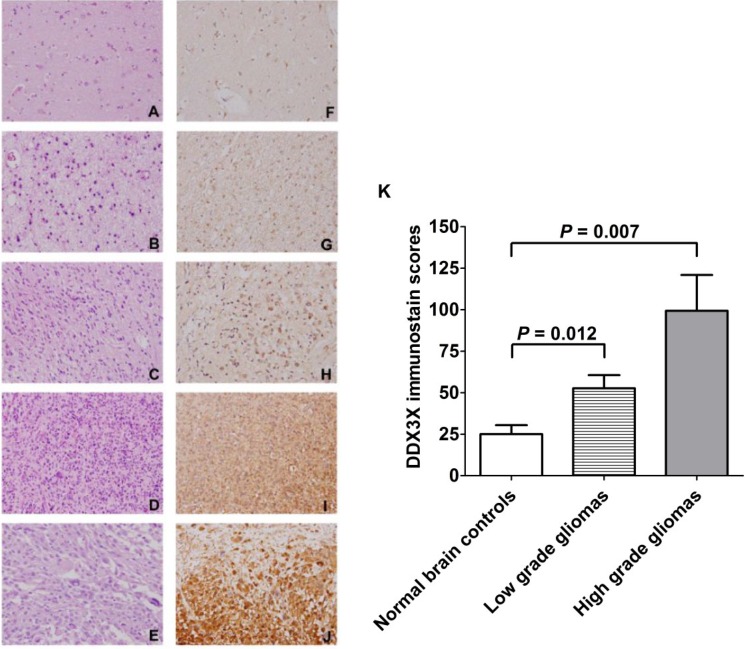
Hematoxylin and eosin staining of non-tumor brain tissue (**A**); pilocytic astrocytoma (**B**); diffuse astrocytoma (**C**); WHO grade III anaplastic astrocytoma (**D**); WHO grade IV glioblastoma multiforme (**E**); and IHC staining of DDX3X in non-tumor brain tissue (**F**); pilocytic astrocytoma (**G**); diffuse astrocytoma (**H**); anaplastic astrocytoma (**I**); and glioblastoma multiforme (**J**). Original magnification ×400; and (**K**) The DDX3X immunostain scores in normal brain tissue, low-grade glioma and high-grade glioma were statistically analyzed. The adjusted *p* value was calibrated between each group.

DDX3X protein production in human gliomas tissues and normal brain tissues was checked by IHC staining of tissue microarray. As shown in Figure 5, DDX3X protein overexpressed in high-grade gliomas (WHO grade IV, and III) compared with low-grade gliomas, or non-tumor brain tissue control. Moreover, the correlation of DDX3X immunostain score in non-tumor brain tissue and gliomas revealed positive correlation (*p* = 0.017), which was analyzed by Pearson Product Method Correlation test (the correlation coefficient (γ) of this analysis is 0.466). Furthermore, as shown in Figure 5K, DDX3X immunostain scores were significantly higher in high-grade gliomas than in normal brain controls (average score, 99.29 *vs.* 25, *p* = 0.007). Finally, DDX3X immunostain scores were also statistically higher in low-grade gliomas (average score, 52.65) than in normal brain control (*p* = 0.012, *p* adjusted by Bonferroni method).

### 2.6. DDX3X Hub Protein and the Protein–Protein Interactions

**Figure 6:**
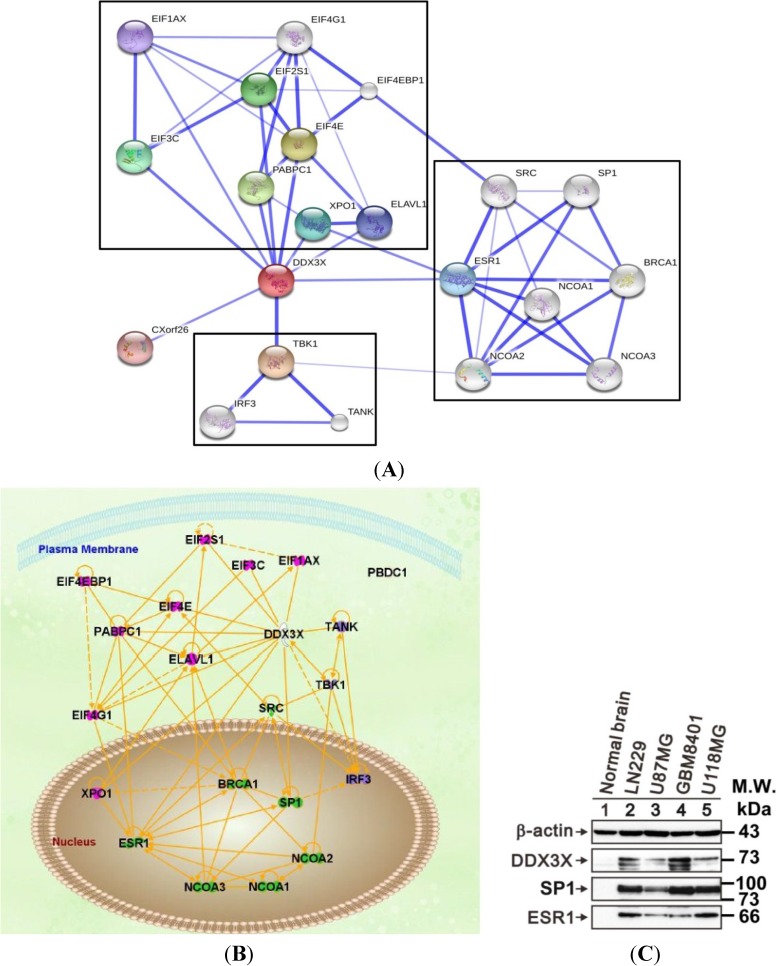
The protein–protein interaction (PPI) network and ingenuity pathway analysis (IPA). (**A**) The PPI network, as shown in the confidence view, created by the STRING database. DDX3X is controlling hub, and black squares show the protein clusters; (**B**) The IPA predicted signaling pathways transcriptionally regulated through DDX3X; and (**C**) The DDX3X-regulated genes including ESR1, and transcription factor SP1 were validated in normal brain tissue and human glioma cell lines. Protein lysates of glioma cell lines including LN229, U87MG, GBM8401 and U118MG were applied to SDS-PAGE and Western blot analysis to quantitate DDX3X, ESR1, and SP1 protein expression. These results are representative of two independent experiments. β-actin served as a loading control.

The protein–protein interaction (PPI) network of DDX3X-regulated oncogenesis was generated using Search Tool for the Retrieval of Interacting Genes/Proteins (STRING) database [20]. The network revealed three PPI clusters linked by DDX3X as a potential hub protein (Figure 6A). Moreover, the Ingenuity pathway analysis (IPA) predicts that DDX3X plays the transcriptional regulation role for ESR1, SP1, XPO1, and serine/threonine-protein kinase TANK-binding kinase 1 (TBK1) to translocate into nucleus in signaling pathways (Figure 6B). Furthermore, the DDX3X-regulated proteins in human gliomas were validated by Western blot analysis. The DDX3X protein highly expresses in human glioma cells also correlates with the protein level of SP1, and ESR1 in human glioma cell lines including LN229, U87MG, GBM8401 and U118MG as compared with normal brain tissue (Figure 6C).

## 3. Discussion

The present studies suggest that DDX3X expression is significantly higher in patients with high-grade gliomas than in those with non-tumor brain controls and low-grade gliomas. High DDX3X expression is also associated with poor survival outcome. Molecular studies including qRT-PCR, Western blotting, and IHC staining further confirmed that DDX3X is over expressed in high-grade gliomas. This is the first study to establish the correlation between DDX3X biomarker with clinical outcome, and WHO pathological grading in human gliomas. Since the therapeutic outcome of GBM is not satisfactory, DDX3X provides one of the candidates for clinicians to choose biomarker to evaluate the survival outcome of patients with GBM stratified by the DDX3X expression in high-grade gliomas.

Western blot analysis of 31 patients with GBM showed high level expression of DDX3 in sixteen patients [17]. There is a statistically significant positive correlation between the levels of DDX3 and transcription factor Snail, which requires DDX3X to express. The fact that HDAC inhibitors also increased Snail expression hints that the action of DDX3X may contribute to the promotion of the progression in human GBM [17]. Inactivation of DDX3X suppressed Snail expression and alternate cell morphology as well as reduced migration and proliferation of HeLa and MCF-7 cell lines. The inactivation of DDX3X gene leads to suppression of Snail through the mechanism of epithelial mesenchymal transition reversal and increased E-cadherin. [17]. DDX3X also regulates DNA damage-induced apoptosis and p53 stabilization in MCF-7, suggesting suppression of DDX3X might leads to apoptosis and increased radiosensitization, which suggests suppression of DDX3X might enhance radiotherapy of human gliomas effectively.

The CD133 expressing glioma cell population plays a crucial role in radioresistance and recurrence of human gliomas [21]. The immunogenic protein DDX3X is shown in high quantity in CD133-expressing tumor cells. The DDX3X primed specific T cells vaccination contributed to defensive and beneficial antitumor immunity [14]. Overexpression of DDX3X in MCF 10A breast cancer cells prompts an epithelial-mesenchymal-like transformation, increased migration, invasion, and anchorage-independent colony formation [11]. However, currently the role of DDX3X in angiogenesis and autophagy is not yet clarified. Further investigations are needed.

The DDX3X has tissue specific differential protein production profiles and plays an oncogenic role in human brain glioma, breast cancer, and cervical cancer. In contrast, DDX3X is downregulated in hepatocellular carcinoma tissue compared to paired adjacent non-tumor tissues [22].

From the protein–protein interactions (PPI) database, STRING, three PPI clusters linked by hub protein DDX3X was found. PPI predicts the protein DDX3X is involved in regulation of the transcriptional signal ESR1, SP1, XPO1, and TBK1 that translocate into nucleus in signaling pathways. Validation of DDX3X-regulated genes found that DDX3X protein highly expresses in human glioma cells and correlates the protein level of SP1, and ESR1. Consistently, DDX3X also statistically associates with ESR1 in breast cancer [12]. ESR1, a known SP1 co-activator, is a nuclear hormone receptor, which is involved in regulation of cellular proliferation and binding to estrogen response element association with DNA-binding transcription factor SP1 with functional synergy [23,24]. Thus DDX3X is a crucial element of the TBK1-dependent innate immune response [25]. Further wet lab approach to validate downstream targets of DDX3X would be another one interesting study.

The limitations of this study include scarcity of human brain gliomas, rare incidence of grade I and low-grade glioma samples, and to pare with non-tumor control for validation of protein level or mRNA expression. For this reason, large-scale analyses of data from two GEO profiles were conducted to determine the role of DDX3X as a pathological grade and survival outcome markers as shown in the results. Using wet lab approaches such as qRT-PCR, Western blotting, and IHC staining consolidate the validation of the data from GEO profile. Moreover, the clinical validity of DDX3X as a true biomarker for unfavorable survival would be tested prospectively, and the underlying mechanism to explain the phenomenon will be conducted in future study. Theoretically, the examination of DDX3X protein expression in several pairs of glioma with different grades is important. However, to get normal brain pairs of glioma from human patients is not always accepted by human study ethics. It is not legal to cut the normal brain from patients for study purposes because it may lead to neurological deficits in patients. Brain surgery is not the same as with other cancers, such as breast, stomach, or colon, which can be resected with safe margin of non-tumor parts to be non-tumor part of pairs of cancer samples. Alternatively, we have examined DDX3X protein expression in four glioma cell lines and commercially available normal brain lysate to confirm the DDX3X protein expression is really higher in glioma than in non-tumor brain control.

## 4. Materials and Methods

### 4.1. Study Ethics and DDX3X Gene Expression in Human Brain Tumor

The institutional review board of Tri-Service General Hospital in Taipei, Taiwan, ROC approved the study (TSGHIRB No: B-102-10). The methodology for analyses of functional genomic databases was as described previously [5,18,19,26,27]. Briefly, 100 sheets of de-linked data (GDS1815/201210_at/DDX3X) on DDX3X mRNA expression, sex, age, pathologic grading, and survival of primary high-grade glioma were obtained from NCBI (Available online: http://www.ncbi.nlm.nih.gov/geo/tools/profileGraph.cgi?ID=GDS1815:201210_at). Twenty-three sheets of data without detailed information on age and survival times were excluded so that 77 sheet were included in the statistical analyses.

An additional database (GDS1962/201211_s_at/DDX3X) containing 180 sheets from 81 patients with grade IV glioma, 19 with grade III glioma, seven with grade II glioma, 23 without tumor (non-tumor control), 38 with grade II oligodendroglioma, and 12 with grade III oligodendroglioma with value on *DDX3X* gene expression obtained from NCBI (Available online: http://www.ncbi.nlm.nih.gov/geo/tools/profileGraph.cgi? ID=GDS1962:201211_s_at) was also included.

### 4.2. RNA Isolation and Quantitative RT-PCR (qRT-PCR)

LN229, U87MG, GBM8401, U118MG, and HeLa cells were maintained in Dulbecco’s modified Eagle’s medium (DMEM) containing 10% fetal bovine serum (FBS), penicillin, and streptomycin. Total RNA was extracted using the EasyPure Total RNA reagent (Bioman, Taipei, Taiwan) according to the manufacturer’s protocol. For cDNA synthesis, 1.0 μg RNA was reverse transcribed (RT) into cDNA using Oligo dT primer with MMLV Reverse Transcriptase (Epicentre Biotechnologies, Madison, WI, USA). The normal brain cDNA was purchased from Origene Technologies (Rockville, MD, USA).

Gene expression was quantified by qRT-PCR and performed in illumina ECO™ Real-Time PCR system. The amplifications were done using IQ^2^ fast qPCR system with ROX (Bio-genesis Technology Inc., Taipei, Taiwan). The relative quantitative gene expression against an internal control, GAPDH was performed using the 2^−ΔΔ*C*t^ method. The primer pairs used were: DDX3X forward, 5′-ATGGCTTGTGCCCAAACAG-3′ and reverse, 5′-CGCCTGGACCATCTGAATAAA-3′ [28] and GAPDH forward, 5′-CTTCATTGACCTCAACTAC-3′and reverse, 5′-GCCATCCACAGTCTTCTG-3′.

### 4.3. Cell Lysate Preparation and Western Blots

Cells were lysed by RIPA buffer (100 mM Tris-HCl, 150 mM NaCl, 0.1% SDS, and 1% Triton-X-100) at 4 °C for 10 min, and the cell lysates were harvested by centrifugation at 15,000 rpm for 10 min to obtain the supernatants. The normal brain lysate were purchased from Origene Technologies. Thirty-microgram cell lysates from each group were applied to 10% sodium dodecyl sulfate polyacrylamide gels electrophoresis. Proteins were transferred onto polyvinyldifluoride membranes (Millipore, MA, USA) and blocked with 5% skim milk in TBST for 1 h at room temperature. The antibodies used are anti-DDX3X (GeneTex, San Antonio, TX, USA), anti-estrogen receptor alpha (ERα) antibody (H-184) (cat. No. Sc-7207; Santa Cruz Biotechnology, Santa Cruz, CA, USA), anti-SP1 antibody (Millipore, Upstate, MA, USA) and β-actin (Santa Cruz Biotechnology, Inc.). Band detection was conducted by enhanced chemi-luminescence and X-ray film (GE Healthcare, Piscataway, NJ, USA).

### 4.4. Immunohistochemical (IHC) Staining

IHC staining of tissue microarray (BS17016; Biomax, Rochester, NY, USA) was conducted according to previous protocol [26,29], incubated with a polyclonal rabbit anti-human DDX3X antibody (N3C2) (1:250 diluted in phosphate buffered saline (PBS); GeneTex) for 1 h at room temperature, washed 3 times (each for 5 min in PBS), incubated with biotin-labeled secondary immunoglobulin (1:100, DAKO, Glostrup, Denmark) for 1 h at room temperature, washed 3 times, and treated with 3-amino-9-ethylcarbazole substrate chromogen (DAKO) at room temperature to visualize peroxidase activity [19]. Sections of breast cancer tissue (known to stain positive for DDX3X) were used as positive control [12].

### 4.5. Evaluation of DDX3X Immunostain Scores

The evaluation of DDX3X immunostain scores has been mentioned by previous study with minor modification [19]. To assess immuno-reactivity in histologic sections, all tissue microarray experiments were repeated twice and the histologic slides were checked and scored by two pathologists concurrently. To evaluate DDX3X immuno-staining scores, the intensity of membranous and cytoplasmic staining was scored as 3 (strong staining), 2 (moderate staining), 1 (weak staining), or 0 (absence of staining). Strong, moderate, and weak cytoplasmic stains were recognized by microscopy with magnification of 10×, 20×, and 40×, respectively. The cut-off value of DDX3X intensity scores was deposit by the magnification of microscope objective lens. The percentage score of tissue staining was calculated. The DDX3X immunostain scores of human gliomas were defined by multiplying the percentage score and the corresponding intensity score.

### 4.6. PPI Network and Signaling Pathways Analysis

Known and predicted protein-protein interactions were analyzed by using Search Tool for the Retrieval of Interacting Genes/Proteins (STRING) database version 10.0 (http://string-db.org) [20]. The prediction of signaling pathway analysis was applied using Ingenuity Pathways Analysis (IPA) software (http://www.ingenuity.com) [30].

### 4.7. Statistical Analysis

The methodology for statistical analysis has been described previously [5]. Combined strategies were used to analyze the two datasets (GDS1815/201210_at/DDX3X and GDS1962/201211_s_at/DDX3X) gained from the GEO profiles. The values of *DDX3X* gene expression in the four WHO pathologic grades and the DDX3X immunostain scores were calculated by single tail test. The Bonferroni method was applied to adjust the *p* value to rule out the possibility of type I error in multi-groups analyses. The Kaplan–Meier method was applied for the overall survival analysis and cohorts of low- *vs.* high-DDX3X expressions were analyzed in high-grade gliomas. Chi-square test was used to analyze survival parameters in patients from WHO grade IV combined with grade III human glioma groups. The cut-off value of DDX3X expression was determined by statistical analysis to get the adequate value. Statistical analyses were performed using the R 3.1.2 software (Available online: http://cran.r-project.org). The figures were drawn using GraphPad Prism 5 software (San Diego, CA, USA). A *p* value less than 0.05 was considered as having statistical significance [5,18].

## 5. Conclusions

RNA Helicase DDX3 expression level positively correlates with WHO pathologic grades of gliomas. Overexpression of DDX3 associates with poor clinical survival outcome. Therefore, DDX3 is a valuable biomarker for poor prognosis in human gliomas.
